# Warfarin use and stroke, bleeding and mortality risk in patients with end stage renal disease and atrial fibrillation: a systematic review and meta-analysis

**DOI:** 10.1186/s12882-016-0368-6

**Published:** 2016-10-21

**Authors:** Jingwen Tan, Shuiqing Liu, Jodi B. Segal, G. Caleb Alexander, Mara McAdams-DeMarco

**Affiliations:** 1 Department of Epidemiology, Johns Hopkins Bloomberg School of Public Health, 615 N. Wolfe St, Baltimore, MD 21205 USA; 2 Center for Drug Safety and Effectiveness, Johns Hopkins Bloomberg School of Public Health, Baltimore, MD USA; 3 Department of Medicine, Johns Hopkins School of Medicine, Baltimore, MD USA; 4 Department of Health Policy and Management, Johns Hopkins Bloomberg School of Public Health, Baltimore, MD USA; 5 Department of Surgery, Johns Hopkins School of Medicine, Baltimore, MD USA

**Keywords:** End stage renal disease, Atrial fibrillation, Anticoagulants, Warfarin

## Abstract

**Background:**

Patients with end stage renal disease (ESRD), including stage 5 chronic kidney disease (CKD), hemodialysis (HD) and peritoneal dialysis (PD), are at high risk for stroke-related morbidity, mortality and bleeding. The overall risk/benefit balance of warfarin treatment among patients with ESRD and AF remains unclear.

**Methods:**

We systematically reviewed the associations of warfarin use and stroke outcome, bleeding outcome or mortality in patients with ESRD and AF. We conducted a comprehensive literature search in Feb 2016 using key words related to ESRD, AF and warfarin in PubMed, Embase and Cochrane Library without language restriction. We searched for randomized trials and observational studies that compared the use of warfarin with no treatment, aspirin or direct oral anticoagulants (DOACs), and reported quantitative risk estimates on these outcomes. Paired reviewers screened articles, collected data and performed qualitative assessment using the Cochrane Risk of Bias Assessment Tool for Non-randomized Studies of Interventions. We conducted meta-analyses using the random-effects model with the DerSimonian - Laird estimator and the Knapp-Hartung methods as appropriate.

**Results:**

We identified 2709 references and included 20 observational cohort studies that examined stroke outcome, bleeding outcome and mortality associated with warfarin use in 56,146 patients with ESRD and AF. The pooled estimates from meta-analysis for the stroke outcome suggested that warfarin use was not associated with all-cause stroke (HR = 0.92, 95 % CI 0.74–1.16) or any stroke (HR = 1.01, 95 % CI 0.81–1.26), or ischemic stroke (HR = 0.80, 95 % CI 0.58–1.11) among patients with ESRD and AF. In contrast, warfarin use was associated with significantly increased risk of all-cause bleeding (HR = 1.21, 95 % CI 1.01–1.44), but not associated with major bleeding (HR = 1.18, 95 % CI 0.82–1.69) or gastrointestinal bleeding (HR = 1.19, 95 % CI 0.81–1.76) or any bleeding (HR = 1.21, 95 % CI 0.99–1.48). There was insufficient evidence to evaluate the association between warfarin use and mortality in this population (pooled risk estimate not calculated due to high heterogeneity). Results on DOACs were inconclusive due to limited relevant studies.

**Conclusions:**

Given the absence of efficacy and an increased bleeding risk, these findings call into question the use of warfarin for AF treatment among patients with ESRD.

## Background

The prevalence of atrial fibrillation (AF) in adults with end stage renal disease (ESRD) is 11.6 % [1], about 11-times higher than the prevalence of AF in the general adult population [2]. Among patients with ESRD and AF, the incidence of stroke is 5.2 per 100 person-years and the incidence of mortality is 26.9 per 100 person-years. These incidences are notably higher than the incidence of stroke (1.9 per 100 person-years) and the incidence of mortality (13.4 per 100 person-years) in patients with ESRD who do not have AF [1].

Anticoagulation therapy, such as warfarin, is commonly prescribed to prevent ischemic stroke and its efficacy is well demonstrated in a meta-analysis of randomized trials and observational studies in patients with chronic kidney disease (CKD) and AF [3]. Another meta-analysis suggested that using warfarin does not significantly increase adverse bleeding outcomes among patients with AF and mild to moderate CKD [4]. Direct oral anticoagulants (DOACs) including dabigatran, rivaroxaban and apixaban are available as alternatives to warfarin therapy for prevention of stroke and systemic thromboembolism in patients with AF without renal impairment. Some data on DOACs suggested a higher efficacy [3] and lower bleeding risk [4] of these agents compared with warfarin in patients with CKD and AF. However, the product labels stated that the use of dabigatran and rivaroxaban should be avoided in patients with severe renal impairment (i.e. creatinine clearance [CrCl] < 30 mL/min). Previous randomized controlled trials have excluded patients with advanced CKD on dialysis and thus there remains a lack of evidence to support the use of warfarin or DOACs in this population. Despite the wealth of evidence of anticoagulation therapy in patients with CKD, the benefits and risks of warfarin and DOACs in patients with ESRD and AF are unclear.

The guidelines for warfarin treatment in patients with ESRD and AF are not uniform. The current American Heart Association/American College of Cardiology/Heart Rhythm Society (AHA/ACC/HRS) guideline recommends warfarin for oral anticoagulation in patients with ESRD and nonvalvular AF who have a CHA_2_DS_2_-VASc score of 2 or greater [5]. The Kidney Disease Improving Global Outcomes (KDIGO) guideline suggests that routine anticoagulation in patients with ESRD and AF for primary prevention of stroke is not indicated because of increased risk for bleeding and lack of systematic evidence for stroke prevention benefit, whereas recommendations for secondary prevention and careful monitoring of all patients receiving dialysis anticoagulation remain valid [6]. Recently published systematic reviews which examined the benefit and risk of warfarin in patients with ESRD and AF were limited to patients on hemodialysis (HD) or peritoneal dialysis (PD) [7, 8], or used an inappropriate measure of association (risk ratio [RR] instead of hazard ratio [HR]) [9]. Because the risk of outcomes may not remain constant over the study period and loss to follow up are common in observational studies, HR is more appropriate for evaluating the effects of warfarin as most observational studies report time-to-event data. Moreover, these studies reported conflicting results regarding the association between warfarin use and stroke outcome: one review reported warfarin use was associated with higher risk of any stroke (RR 1.50, 95 % CI: 1.13–1.99) [9] while other reviews reported a lack of association between warfarin use and stroke [7, 8, 10].

Therefore, we expanded the population to stage 5 CKD, HD, and PD and conducted a systematic review and meta-analyses on the benefits and risks of warfarin use. We used appropriate analytic tools such as the Cochrane Risk of Bias Assessment Tool for Non-randomized Studies of Interventions [11] for qualitative assessment and the Knapp-Hartung methods [12] for quantitative assessment. The objective of this study was to review and summarize the associations between warfarin use and stroke outcomes, bleeding outcomes and all-cause mortality, as compared to no warfarin use, aspirin or DOACs, among patients with ESRD and AF.

## Methods

### Search strategy

We performed the systematic review and meta-analysis in adherence to the Preferred Reporting Items for Systematic Reviews and Meta-Analyses (PRISMA) guidelines. First, we searched PubMed, Embase and the Cochrane Central register using synonyms and variations of the following search terms without language or date restrictions: “end stage renal disease”, and “atrial fibrillation” and “anticoagulants or warfarin”. We used a combination of controlled vocabulary (e.g. MeSH and Emtree), free-text words (i.e. words appearing in the title, abstract or keywords of a database entry), and truncated terms as appropriate for each database (Appendix). All databases were searched from their start date to February 10, 2016. In addition to the electronic database searches, we hand-searched the reference lists of review articles, relevant studies and clinical practice guidelines.

### Study selection

We searched for published randomized controlled trials (RCTs) and quasi-RCTs (RCTs in which allocation to treatment was obtained by alternation, use of alternate medical records, date of birth or other predictable methods), and observational studies which examined the benefits and risks of warfarin in patients with ESRD and AF. We included studies of at least 10 patients with ESRD (HD, PD, stage 5 CKD i.e. GFR < 15 mL/min/1.73 m^2^) and with pre-existing or newly diagnosed AF (all types). We also included studies with broader study populations (i.e. CKD) if they reported outcomes separately for participants with ESRD. We included studies which compared warfarin use with placebo, no treatment or other antithrombotic agents (e.g. aspirin, dabigatran, rivaroxban, apixaban). Studies needed to report quantitative data on the risk for any of the following outcomes: all-cause stroke (any stroke i.e. including ischemic stroke, hemorrhagic stroke, systematic thromboembolism and transient ischemic attacks; ischemic stroke), all-cause bleeding (any bleeding, major bleeding, gastrointestinal bleeding) or all-cause mortality. We only reviewed full articles because conference abstracts would not provide the details necessary for qualitative and quantitative assessments.

### Data collection

Two reviewer authors (JT, SL) independently conducted abstract screening and selected relevant studies for data abstraction according to the inclusion/exclusion criteria above. We used a web-based systematic review software DistillerSR (Evidence Partners, Ottawa, Canada) to document the article screening process and to develop standardized data collection forms. For each study, we abstracted bibliographic information (first author, publication year); general information (location of study, sample size, number of treatment groups, number of participants); participant characteristics (age, gender, history of stroke and bleeding); interventions (treatment groups); outcomes (definition, analytic method, crude event data, adjusted risk estimates (HR) and their 95 % CIs); and study quality. We also contacted the corresponding authors of four included studies [13–16] to obtain missing outcome data. We used the Cochrane Risk of Bias Assessment Tool for Non-Randomized Studies of Interventions (ACROBAT-NRSI) [11] to assess the risk of bias because it was designed specifically for non-randomized studies that evaluate effectiveness of interventions. We rated the risk of bias on seven domains at the study level, and rated the overall risk of bias based on the domain with the highest risk of bias. Discrepancies in study selection and data collection were resolved by the two reviewers through discussions and consensus.

### Data analysis

We assessed the clinical and methodologic heterogeneity in participant characteristics (i.e. ESRD status, age, gender, comorbidities, prevalent vs. incident warfarin users) and assessments of outcomes (i.e. outcome definitions and analytic methods). We used the Cochran Q test, which follows a Chi-square distribution with n-1 of freedom, with an alpha of < 0.10 to assess the presence of statistical heterogeneity between studies. We also calculated the I^2^ statistic, which ranges between 0 and 100 %, to determine the proportion of between group variability that is attributable to heterogeneity rather than chance [17]. If there was evidence for considerable heterogeneity (i.e. I^2^ ≥ 80 %), we displayed the risk estimates in a forest plot but did not calculate the overall risk estimates. Otherwise, we conducted meta-analyses using the random effects model with the DerSimonian-Laird estimator [18] and the Knapp-Hartung approach [12], where appropriate, using Stata 14.0 (StataCorp, College Station, TX).

### Sensitivity analysis

We performed pre-specified sensitivity analyses where studies with prevalent warfarin users and studies with low methodological quality were excluded from meta-analyses. We assessed the presence of publication bias using funnel plots with the natural log of HR plotted on the y-axis and the standard error of natural log of HR plotted on the x-axis. We also tested the presence of small-study effects using the Egger’s test [19]. We conducted meta-regressions with the Knapp-Hartung approach to evaluate the impact of study quality (moderate/high risk of bias vs critical risk of bias), patient population (HD only vs mixed ESRD population), and study design (studies including incident warfarin users only vs. studies including prevalent and incident warfarin users) on stroke outcome, bleeding outcome or mortality.

## Results

### Description of included studies

**Fig. 1:**
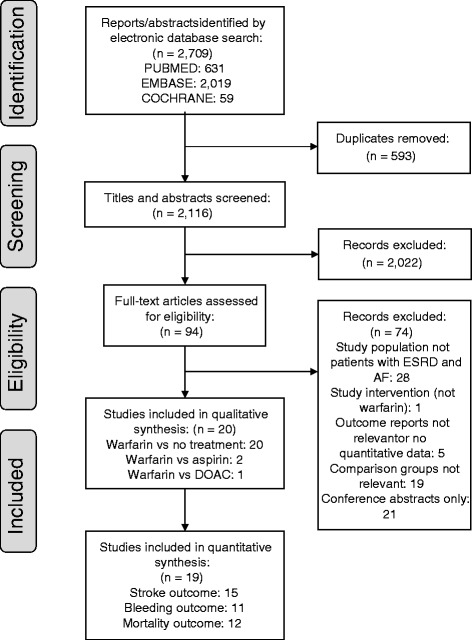
PRISMA flow diagram of study selection for systematic review

We identified 2709 references from the electronic database search; no additional references were identified from hand searching. After removing 593 duplicate references and excluding 2022 references in titles and abstracts screening, we did a full-text review of 94 references. After excluding another 74 references, we included 20 articles for qualitative and quantitative assessment (Fig. 1).

All 20 investigations were observational cohort studies examining the outcomes of warfarin use in patients with ESRD and AF. Nineteen studies compared warfarin use to no warfarin use, while two studies also compared warfarin use to aspirin [20, 21] and one study compared warfarin use to dabigatran and rivaroxaban [20]. A total of 56,146 patients with ESRD and AF, including 34,840 on HD, 315 on PD, 610 with stage 5 CKD, and 20,381 mixed ESRD population, were included in these studies. These studies included a median of 690 (interquartile range [IQR] 204–3012) patients with ESRD and AF, and had a median duration of 7.0 (IQR 2.9–9.4) years. Eight studies were based on administrative claims or national/regional registry data [13, 22–28], and they generally were longer and larger studies. Twenty studies included participants with a mean age above 60 years old, including two studies that were limited to older adults above 65 years old [22, 27]. Nine studies examined effects of warfarin use between incident warfarin users and nonusers [20, 22–29], whereas the other 11 studies compared prevalent warfarin users with nonusers (Table 1, Appendix Table 3).

**Table 1:** Characteristics of warfarin studies in patients with end stage renal disease and atrial fibrillation

Author Year	Setting	Study duration (years)	Study population	Study groups	Number of patients with ESRD and AF	% Female	Mean age (SD) (years)	% With stroke/TIA/TE history	% With bleeding history
Chan 2009 [30]	US, Fresenius clinics	1.6	Patients with incident HD, pre-existing AF	T (total)	1671	NR	NR	NR	NR
				W (warfarin)	746	NR	NR	NR	NR
				C (no warfarin)	925	NR	NR	NR	NR
Lai 2009 [32]	US, single center	2.6	All patients with CKD (HD and GFR < 15 mL/min/1.73 m^2^) and pre-existing non-valvular AF, includes prevalent warfarin users	T (total)	245	NR	NR	NR	NR
				W (warfarin)	129	NR	NR	NR	NR
				C (no warfarin)	96	NR	NR	NR	NR
Wizemann 2010 [33]	International consortium	8	Patients with HD who had pre-existing or newly developed AF, includes prevalent warfarin users	T (total)	3245	NR	NR	NR	NR
				W (warfarin)	509	NR	NR	NR	NR
				C (no warfarin)	2736	NR	NR	NR	NR
Winkelmayer 2011 [22]	US, New Jersey, Pennsylvania Medicare claims	22	All patients with incident dialysis ≥ 66 years who had first hospitalization with a primary or secondary discharge diagnosis of AF	T (total)	2313	NR	NR	NR	NR
				W (warfarin)	249	57.4	68.6 (12.1)	NR	6.8
				C (no warfarin)	2064	57.5	70.1 (11.9)	NR	16.2
Olesen 2012 [23]	Denmark, national registry	12	All patients discharged from the hospital with a diagnosis of non-valvular AF, receiving RRT	T (total)	901	33.6	66.8 (11.7)	14.8	15.2
				W (warfarin only)	178	NR	NR	NR	NR
				C (no warfarin)	723	NR	NR	NR	NR
Khalid 2013 [34]	US, multi-center	6	Patients who were started on warfarin in the last year and re-started warfarin for atrial fibrillation after a gastrointestinal bleed	T (total)	96	31.3	77.2 (10.6)	52.1	21.2
				W (restarted warfarin)	34	NR	NR	NR	NR
				C (did not restart warfarin)	62	NR	NR	NR	NR
Wakasugi 2014 [29]	Japan, multi-center	3	Patients aged ≥ 20 years with ESRD requiring HD and pre-existing chronic sustained AF, includes prevalent warfarin users	T (total)	60	NR	NR	NR	NR
				W (warfarin)	28	43	67.8 (9.4)	14	NR
				C (no warfarin)	32	28	68.4 (8.5)	36	NR
Bonde 2014 [24]	Denmark, national registry	15	Incident non-valvular AF discharge, receiving RRT, stratified by CHA_2_DS_2_-VASc score	T (total)	1142	35.03	66.77 (12.03)	16.37	17.51
				W (warfarin)	260	NR	NR	NR	NR
				C (no warfarin)	882	NR	NR	NR	NR
Carrero 2014 [25]	Sweden, national registry	7	Survivors of acute myocardial infarction, history of AF or AF diagnosis in hospital, eGFR ≤ 15 ml/min/173 m^2^	T (total)	478	NR	NR	NR	NR
				W (warfarin)	66	37.9^a^	78^a^ (NR)	28.8^a^	12.1^a^
				C (no warfarin)	412	38.8^a^	77^a^ (NR)	26.5^a^	22.8^a^
Chen 2014 [26]	Taiwan, national registry	4.12	Adult (≥18 years) patients with ESRD, receiving RRT, pre-existing non-valvular AF	T (total)	3277	NR	NR	NR	NR
				W (warfarin)	294	58.5	NR	NR	NR
				C (no warfarin)	2983	53.7	NR	NR	NR
Friberg 2014 [13]	Sweden, national registry	2.1	Any inpatient diagnosis of non-valvular AF, receiving RRT, includes prevalent warfarin users	T (total)	13435	35.7	78.4 (10.3)	24.6	30.5
				W (warfarin)	3766	NR	NR	NR	NR
				C (no warfarin)	9669	NR	NR	NR	NR
Shah 2014 [27]	Canada, Quebec & Ontario regional claims	9	Patients aged ≥ 65 years admitted to a hospital with a primary or secondary diagnosis of AF who underwent > = 3 dialysis procedure within the 12 months preceding AF	T (total)	1626	NR	NR	NR	NR
				W (warfarin)	756	39	75.3 (8.1)	6	9
				C (no warfarin)	870	39	75.1 (8.5)	5	16
Genovesi 2015 [31]	Italy, multi-center	2	Patients with HD, pre-existing paroxysmal, persistent or permanent AF, includes prevalent warfarin users	T (total)	290	40.0	NR	14.8	19.7
				W (warfarin)	134	35.8	NR	15.7	11.9
				C (no warfarin)	156	43.6	NR	14.1	26.3
Chan KE 2015 [20]	US, Fresenius clinics	4	Patients with chronic HD, pre-existing AF	T (total)	14607	NR	NR	NR	NR
				W (warfarin)	8064	38.8	70.6 (11)	12.7	3.3
				A (aspirin)	6018	42.7	71.7 (11)	14.3	0.7
				D (dabigatran)	281	40.8	68.4 (12)	12.5	4.1
				R (rivaroxaban)	244	39.5	66.9 (12)	16.0	4.2
Chan PH 2015 [21]	China, single center	14.5	Patients with PD who had a diagnosis of AF treated in two hospitals, exclude HD or CKD stage 5 not on RRT, includes prevalent warfarin users	T (total)	271	NR	NR	NR	NR
				W (warfarin)	67	41.8	69.5 (9.5)	17.9	1.5
				A (aspirin)	86	41.9	73.0 (10.0)	25.6	4.7
				C (no antithrombotic therapy)	118	38.1	69.4 (12.7)	10.2	0.8
Shen 2015 [28]	US, USRDS national registry	4	All patients with HD who had a new diagnosis of AF based on 1 inpatient or 2 outpatient diagnosis codes	T (total)	12284	NR	NR	NR	NR
				W (warfarin)	1838	50.3	61.2 (12.4)	NR	NR
				C (no warfarin)	10446	51.3	62.1 (13.6)	NR	NR
Wang 2015 [14]	New Zealand, single center	9	Patients with ESRD commenced on long-term dialysis at a hospital who had pre-existing or developed AF, includes prevalent warfarin users	T (total)	141	38.3	61.2 (11.3)	NR	19.1
				W (warfarin)	59	39.0	59.8 (10.5)	NR	16.9
				C (no warfarin)	82	37.8	62.1 (11.8)	NR	20.7
Yodogawa 2015 [35]	Japan, single center	9.5	Patients aged ≥ 20 years with AF and ESRD requiring maintenance HD, includes prevalent warfarin users	T (total)	84	30	NR	5	6
				W (warfarin)	30	20	69.5 (10.7)	10	3
				C (no warfarin)	54	35	70.4 (10.2)	2	7
Findlay 2016 [15]	UK, single center	7	Adult patients receiving hemodialysis, exclude those treated for acute kidney injury, includes prevalent warfarin users	T (total)	293	NR	NR	NR	NR
				W (warfarin)	118	NR	NR	NR	NR
				C (no warfarin)	175	NR	NR	NR	NR
Tanaka 2016 [16]	Japan, multi-center	2.5	Patients with ESRD with dialysis initiation who became stable and were discharged from hospital with or without AF, includes prevalent warfarin users	T (total)	93	37.6	NR	NR	NR
				W (warfarin)	46	26.1	73.6 (8.5)	19.6	6.5
				C (no warfarin)	47	34.0	70.7 (12.1)	8.5	0.0

*AF* atrial fibrillation, *HD* hemodialysis, *PD* peritoneal dialysis, *CKD* chronic kidney disease, *ESRD* end stage renal disease, *RRT* renal replacement therapy, *NR* not reported

*A* all relevant patients with ESRD and AF included in study, *T* patients with ESRD and AF in the treatment group, *C* patients with ESRD and AF in the comparison group

^a^ Data were abstracted from the online supplement

### Risk of bias assessment

Due to the observational nature of cohort studies, all 20 included studies had at least an overall rating of moderate risk of bias: 5 studies were rated as moderate [22, 25–28]; 7 studies were rated as serious [13, 14, 20, 23, 24, 30, 31]; 8 studies were rated as having a critical risk of bias [14–16, 21, 29, 32–35] (Table 2, Appendix Table 4).

**Table 2:** Quality assessment of warfarin studies in patients with end stage renal disease and atrial fibrillation

Study	Overall Risk of Bias	Bias due to confounding	Bias in selection of participant into the study	Bias in measurement of interventions	Bias due to departures from intended interventions	Bias due to missing data	Bias in measurement of outcomes	Bias in selection of the reported results
Chan 2009 [30]	Serious	Moderate	Serious	Low	Low	Low	Low	Moderate
Lai 2009 [32]	Critical	Critical	Serious	Serious	Serious	No info	Low	Moderate
Wizemann 2010 [33]	Critical	Serious	Critical	Moderate	No info	No info	Moderate	Serious
Winkelmayer 2011 [22]	Moderate	Moderate	Low	Low	Low	Low	Low	Moderate
Olesen 2012 [23]	Serious	Serious	Low	Low	Serious	Low	Low	Moderate
Khalid 2013 [34]	Critical	Critical	Critical	Moderate	Serious	No info	Low	Moderate
Wakasugi 2014 [29]	Critical	Critical	Serious	Low	Low	No info	Low	Moderate
Bonde 2014 [24]	Serious	Serious	Low	Moderate	Serious	Low	Low	Serious
Carrero 2014 [25]	Moderate	Moderate	Low	Low	Low	Low	Low	Moderate
Chen 2014 [26]	Moderate	Moderate	Low	Low	Low	Low	Low	Moderate
Friberg 2014 [13]	Serious	Moderate	Serious	Low	Low	Low	Low	Moderate
Shah 2014 [27]	Moderate	Moderate	Low	Low	Low	Low	Low	Moderate
Genovesi 2015 [31]	Serious	Moderate	Serious	Low	Low	No info	Low	Moderate
Chan KE 2015 [20]	Serious	Moderate	Low	Low	Serious	Low	Low	Moderate
Chan PH 2015 [21]	Critical	Serious	Critical	Low	Serious	Low	Moderate	Moderate
Shen 2015 [28]	Moderate	Moderate	Low	Low	Low	Low	Low	Moderate
Wang 2015 [14]	Critical	Serious	Critical	Low	Serious	No info	Moderate	Serious
Yodogawa 2015 [35]	Critical	Serious	Critical	Moderate	No info	Low	Moderate	Moderate
Findlay 2016 [15]	Critical	Critical	Critical	No info	Critical	No info	Moderate	Moderate
Tanaka 2016 [16]	Critical	Critical	Critical	Low	Critical	Low	Moderate	Moderate

### Association of warfarin with stroke, bleeding and mortality

**Fig. 2:**
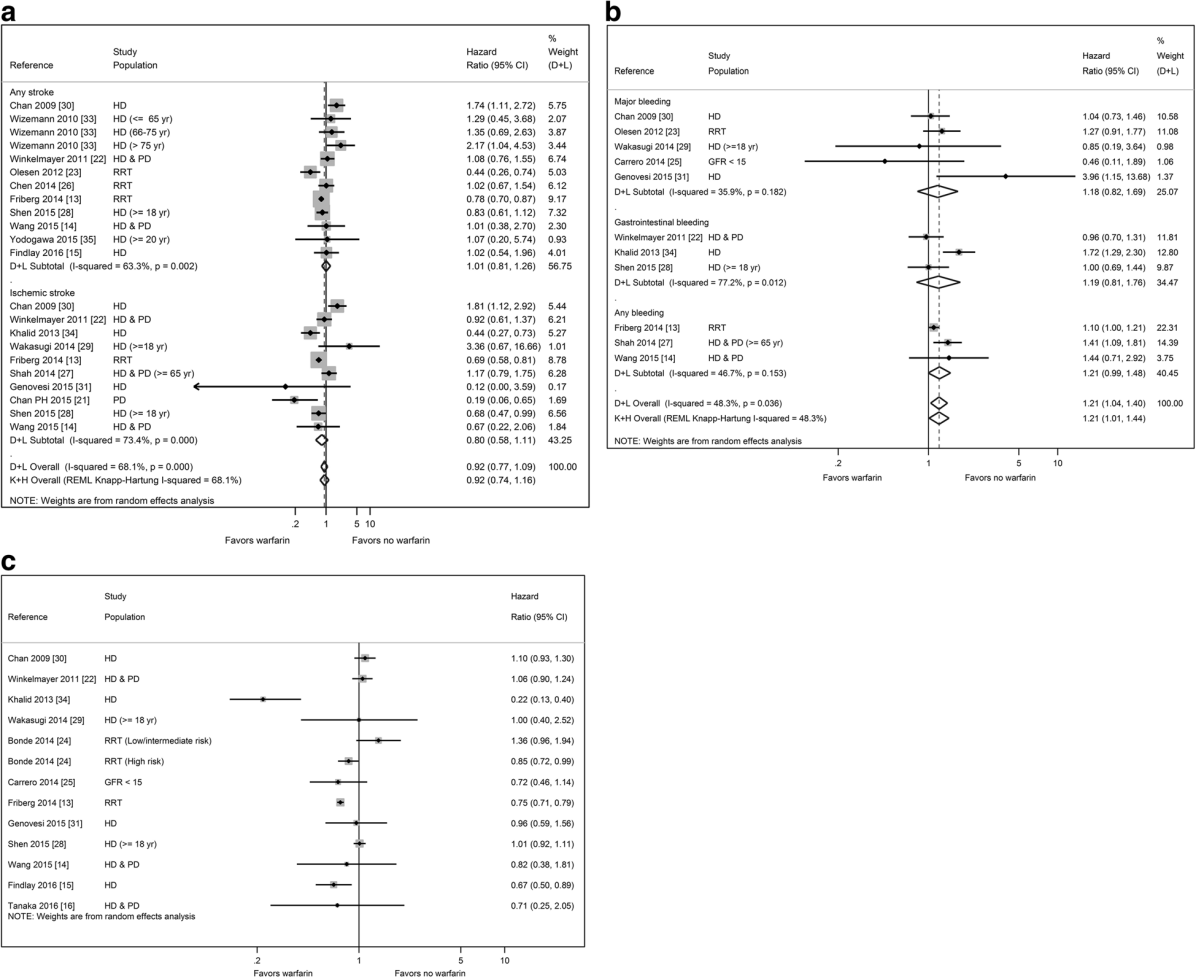
**a** Meta-analysis of stroke outcome in patients with end stage renal disease and atrial fibrillation by warfarin use. **b** Meta-analysis of bleeding outcome in patients with end stage renal disease and atrial fibrillation by warfarin use. **c** Forest plot of mortality in patients with end stage renal disease and atrial fibrillation by warfarin use

The meta-analyses of warfarin use included 15 studies that examined all-cause stroke (I^2^ 68.1 %), 11 studies that examined all-cause bleeding (I^2^ 48.3 %), and 12 studies that examined all-cause mortality (I^2^ 85.7 %) and reported HRs as outcome measures (Fig. 2a-c). Warfarin use was not statistically associated with reduction in all-cause stroke (HR 0.92, 95 % confidence interval [CI] 0.74–1.16) (Fig. 2a), and was not associated with any stroke (HR 1.01, 95 % CI 0.81–1.26) or ischemic stroke (HR 0.80, 95 % CI 0.58–1.11).

By contrast, there was a positive and statistically significant association between warfarin use and all-cause bleeding (HR 1.21, 95 % CI 1.01–1.44) (Fig. 2b). Warfarin use was not associated with major bleeding (HR 1.18, 95 % CI 0.82–1.69) or gastrointestinal bleeding (HR 1.19, 95 % CI 0.81–1.76). While there was a trend towards increased risk of any bleeding, the association was not significant (HR 1.21, 95 % CI 0.99–1.48).

Finally, there was high statistical heterogeneity among the 12 studies (I^2^ = 85.7 %) that examined all-cause mortality, and thus we did not calculate an overall risk estimate (Fig. 2c). Most studies showed non-significant results except for 4 studies that found lower risk of mortality associated warfarin use [13, 16, 24, 34].

### Sensitivity analysis

In the sensitivity analysis in which we excluded 11 studies with prevalent warfarin users, the overall risk estimates were 0.88 (95 % CI 0.65–1.18) for all-cause stroke, 1.14 (95 % CI 0.88–1.47) for all-cause bleeding, and 0.99 (95 % CI 0.83–1.17) for mortality respectively (Appendix Figure 4a–c). In the analysis which we included only studies with prevalent users, the results were consistent with the aforementioned sensitivity analysis: the overall risk estimates were 0.99 (95 % CI 0.69–1.42) for all-cause stroke, 1.31 (95 % CI 0.91–1.87) for all-cause bleeding, 0.72 (HR 0.47–1.11) for all-cause mortality. In the sensitivity analysis in which we excluded studies with critical risk of bias and low methodological quality (Appendix Figure 5a–c), the results were not statistically significant (all-cause stroke: HR 0.91, 95 % CI 0.73–1.14; all-cause bleeding: HR 1.13, 95 % CI 0.99–1.28; pooled risk estimate for mortality not calculated due to heterogeneity).

**Fig. 3:**
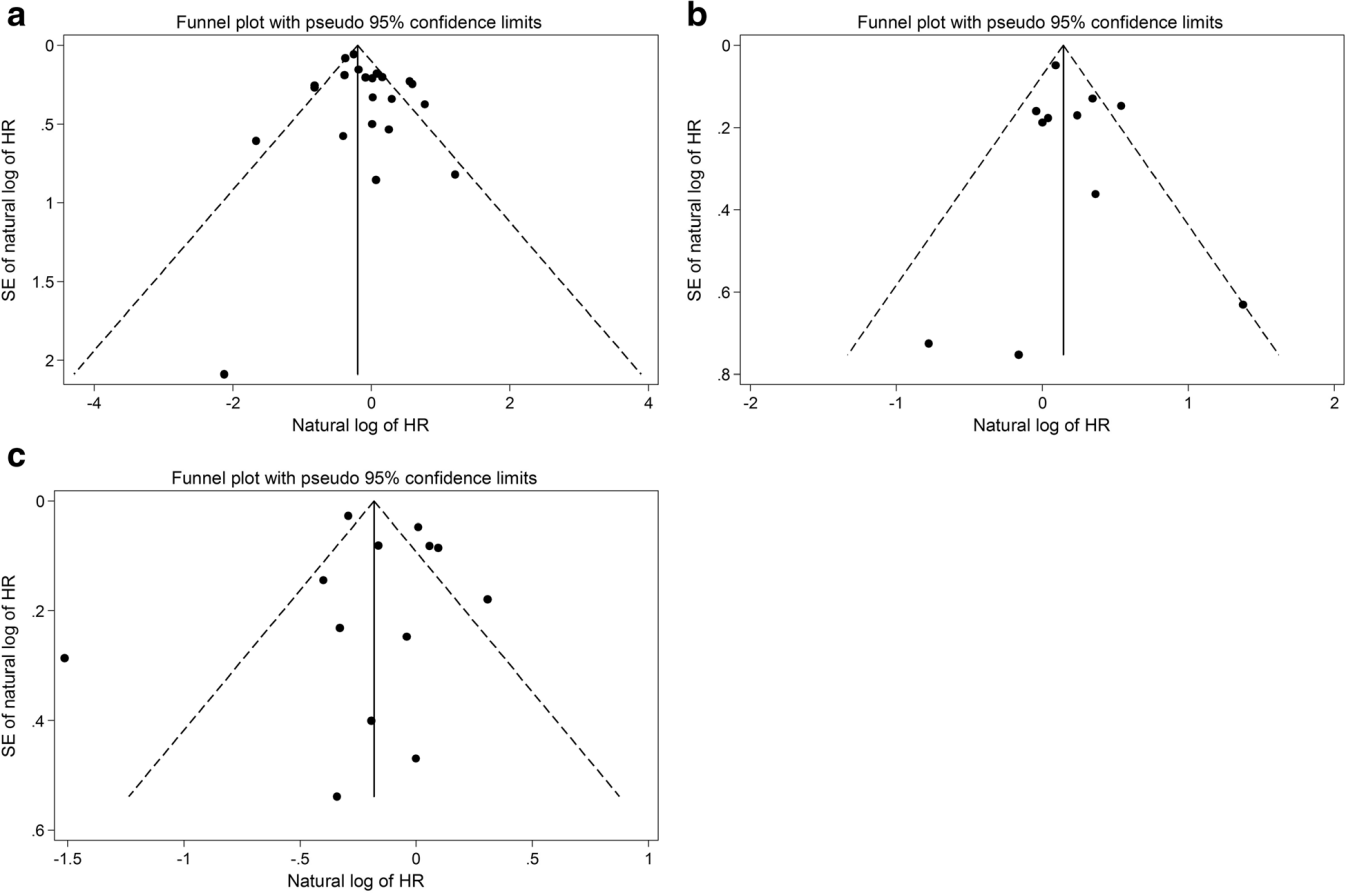
**a** Funnel plot of stroke outcome in patients with end stage renal disease and atrial fibrillation. **b** Funnel plot of bleeding outcome in patients with end stage renal disease and atrial fibrillation. **c** Funnel plot of mortality in patients with end stage renal disease and atrial fibrillation

In the meta-regression, we did not find significant impact of study characteristics on outcomes except for study population in the analysis of all-cause stroke outcome. Compared to studies that included mixed ESRD population, studies including only patients on HD reported higher association with all-cause stroke outcome (OR 5.83, 95 % CI 1.22–27.98; *P* = 0.03). In the funnel plots, we did not observe obvious asymmetry in the funnel plots for all three outcomes (Fig. 3a–c). Statistical tests for small-study effects were not statistically significant for the three outcomes (*P* = 0.21, 0.51 and 0.68 for all-cause stroke, all-cause bleeding and all-cause mortality respectively).

## Discussion

We conducted a systematic review and meta-analyses of 20 observational studies examining the benefits and risks of warfarin use among patients with ESRD and AF. Meta-analyses provided no evidence to suggest associations between warfarin use and all-cause stroke (HR 0.92, 95 % CI 0.74–1.16) among these patients. By contrast, warfarin use was associated with a significantly increased risk of all-cause bleeding (HR 1.21, 95 % CI 1.01–1.44). There were insufficient data with good quality to estimate the association between warfarin use and mortality.

We did not evaluate hemorrhagic stroke in the meta-analyses because only two studies reported hemorrhagic stroke as separate outcomes (Appendix Table 4) [22, 30]. Chan et al. reported that warfarin use was significantly associated with increased risk of hemorrhagic stroke (HR 2.22, 95 % CI 1.01–4.91) [30], and Winkelmayer et al. also reported that warfarin use was associated with hemorrhagic stroke (HR 2.38, 95 % CI 1.15–4.96) [22].

We attempted to evaluate warfarin use vs aspirin or DOACs which was not examined in previously published systematic reviews and meta-analyses, but there were not enough studies to draw conclusions regarding these comparisons. For the two studies that examined ischemic stroke outcomes comparing warfarin vs aspirin, one study showed significant increased risk from warfarin (unadjusted rate ratio (RR) 1.23, 95 % CI 1.01–1.52) [20] whereas another study showed a significant reduced risk (adjusted HR: 0.16, 95 % CI 0.04–0.66) [21]. Warfarin was associated with significantly increased risk of major bleeding compared to aspirin (adjusted RR 1.28, 95 % CI 1.19–1.39) [20]. On the other hand, dabigatran (RR 1.48, 95 % CI 1.21–1.81) and rivaroxaban (RR 1.38, 95 % CI 1.02–1.83) were associated with higher risk of hospitalization or death from bleeding when compared with warfarin [20]. In terms of stroke, the authors noted that there were too few events in the study to detect meaningful differences.

Compared to previously published systematic reviews [7–10], our review included several studies recently published [15, 16, 35] and expanded the study population to include PD [21] and stage 5 CKD [25], which were not included in these reviews [7–9, 36]. Our pooled estimate of ischemic stroke and bleeding outcomes were consistent in direction and magnitude with those reported by Li et al., Liu et al. and Dahal et al. [7, 8, 10]. On the other hand, Lee et al. found that warfarin use was associated with increased risk of any stroke (RR 1.50, 95 % CI 1.13–1.99) [9], but our result was not significant (HR 1.01, 95 % CI 0.81–1.26) because we included eight more studies in our meta-analysis [13–15, 23, 26, 28, 31, 35] and abstracted a less extreme risk estimate from one of the studies [9, 30]. We would like to point out that we abstracted the results from the intention-to-treat analysis from Shen et al. for the meta-analyses [28], whereas other systematic review abstracted the results from the as-treated analysis from Shen et al. [8]. Such difference did not change the general inferences about the lack of association between warfarin use and stroke and the increased risk of bleeding outcome.

Warfarin acts by inhibiting the synthesis of vitamin K-dependent clotting factors, and its anticoagulation effect is influenced by possible interactions between drugs or foods and warfarin [37]. The effectiveness of warfarin use for stroke prevention is crucially dependent on the quality of anticoagulation therapy, which can be monitored by international normalized ratio (INR) and time in therapeutic range (TTR). Only 5 of the included studies discussed the influence of INR or TTR on the outcome results [13, 20, 30–32]. Patients with suboptimal warfarin management (e.g. warfarin users who did not receive INR monitoring [30] or patients with TTR < 60 % [13]) have the highest risk for stroke and thromboembolism. Increasing baseline INR level in warfarin users was positively associated with new stroke [30]. On the other hand, patients with CKD and AF treated with warfarin to maintain an INR between 2.0 and 3.0 had a significant reduction in thromboembolic stroke [32]. Higher TTR, an indicator for good warfarin management, had protective effect against bleeding risk [31]. These results highlighted the difficulty in achieving optimal warfarin management in patients with ESRD and AF, which could help explain the heterogeneous outcomes of warfarin use in this population.

Our review has several strengths including our conduct of a comprehensive search in multiple electronic databases with the application of rigorous qualitative and quantitative assessment. We performed our qualitative assessment using the recently developed Cochrane Risk of Bias Assessment Tool for Non-Randomized Studies of Interventions, which was designed specifically for non-randomized studies that compare the health effects of two or more intervention [11], and unlike the Newcastle-Ottawa scale, does not require modification for use in reviews of effectiveness of interventions [38]. We conducted our quantitative assessment using the Knapp-Hartung method based on small-sample adjustments [12], which provided more accurate confidence limits than the DerSimonian-Laird estimator and has been advocated as an alternative method for meta-analysis with a limited number of studies [39, 40]. In addition, we were able to obtain missing outcome data from four study authors [13–16], and thus examined a greater number of studies than previous authors.

Our report also has several limitations. First, we observed high heterogeneity (I^2^ = 85.7 %) in all-cause mortality across the 12 studies [13–16, 22, 24, 25, 28–30, 34], which limited our ability to estimate a pooled risk estimate. In the sensitivity meta-analysis of all-cause mortality using studies that only included incident warfarin users [22, 24, 25, 28], the pooled risk estimate was not statistically significant (HR 0.99, 95 % CI 0.79–1.22). Second, we were not able to conduct meta-analysis on the association between warfarin use and hemorrhagic stroke or on the comparison between warfarin and aspirin or DOACs because there were insufficient studies available on this topic. Finally, as with all systematic review and meta-analyses, our results were limited by the quality of the available studies for inclusion. Although all included studies reported warfarin use in patients with ESRD and AF, we could not confirm that such use was indicated for AF treatment because the included studies did not report such information. We could not verify that the ischemic outcomes reported in the included studies were confirmed by imaging, since several studies were based on administrative claims or registry data [13, 22, 27, 28].

We observed substantial clinical and methodological heterogeneity in the studies we examined with respect to participant characteristics, study conduct and outcome assessment. Study population seems to have significant impact on the association between warfarin use and all-cause stroke outcome as evidenced in the meta-regression. Compared to 9 studies that included patients on HD only, 6 studies that included mixed ESRD population [13, 14, 22, 23, 26] or patients on PD only [41] reported lower association between warfarin use and all-cause stroke. This may reflect heterogeneous treatment effects among subgroups of patients with ESRD and requires further investigation. Although meta-regression did not show significant impact on outcomes due to study quality or study design, these characteristics helped explain the heterogeneity observed in the included studies. A majority of the included studies had serious or critical risk of bias, particularly in the bias due to confounding [14–16, 21, 23, 24, 29, 32–35], bias in selection of participants [13, 20, 29, 31–34], and bias due to departures from intended interventions domains [13, 15, 16, 20, 21, 23, 24, 31, 32, 34, 42]. While all studies attempted to control for confounding bias by covariate adjustment or propensity score adjustment/matching except for one [15], there may be inherent confounding bias due to unobserved covariates, residual confounding or unsuccessful adjustment. Studies that included prevalent [13, 15, 16, 21, 29, 31–35, 42], rather than new warfarin users, could introduce selection bias because the effect measure was weighted toward prevalent users who had survived the early events [43]. This would underestimate the events that occur early among prevalent users when the risk of treatment-related outcome varies with time [44]. Patients that started on warfarin could discontinue the therapy and thus switched over to the non-use group, leading to bias due to departures from intended interventions [28].

## Conclusions

Despite the degree of heterogeneity across studies and the bias in selected studies, our study showed that warfarin use was not associated with a lower risk of ischemic stroke, consistent with recent studies [7–9], and was associated with a significant higher risk for bleeding [7–10, 36] among patients undergoing HD. There was insufficient evidence with good quality to estimate the association between warfarin use and hemorrhagic stroke or mortality. Given the limitations of observational studies described above, large randomized controlled trials involving patients with ESRD and AF may be warranted to definitively evaluate the benefits and risks of warfarin. However, we recognize that such study may be too costly to be carried out, so high-quality observational studies are necessary to address the clinical decision dilemma regarding warfarin use in this population.

## Figures and Tables

**Fig. 4:**
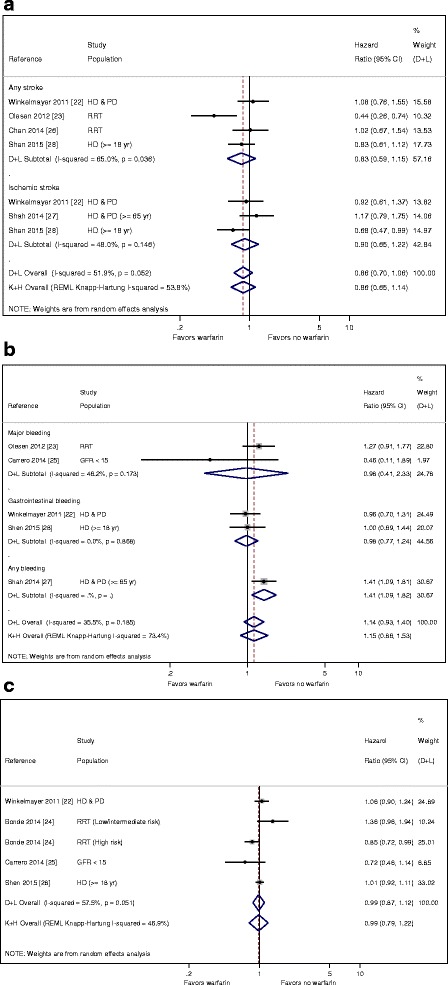
**a** Sensitivity meta-analysis: stroke outcome by warfarin use, excluding studies with prevalent warfarin users. **b** Sensitivity meta-analysis: bleeding outcome by warfarin use, excluding studies with prevalent warfarin users. **c** Sensitivity meta-analysis: mortality by warfarin use, excluding studies with prevalent warfarin users

**Fig. 5:**
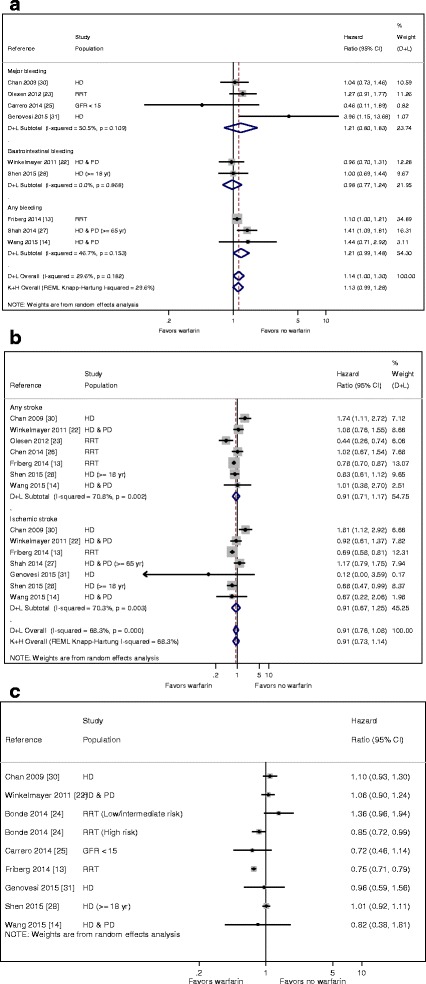
**a** Sensitivity meta-analysis of on stroke outcome by warfarin use, excluding studies with critical risk of bias. **b** Sensitivity meta-analysis of on bleeding outcome by warfarin use, excluding studies with critical risk of bias. **c** Sensitivity meta-analysis of on mortality outcome by warfarin use, excluding studies with critical risk of bias

## Appendix

Search strategies in PubMed, Embase and Cochrane Library

### PubMed

#1 kidney failure, chronic [mh] OR renal insufficiency, chronic [mh] OR renal replacement therapy [mh] OR renal dialysis [mh] OR kidney transplantation [mh]

OR kidney disease [tw] OR renal disease [tw] OR kidney failure [tw] OR renal failure [tw] OR kidney dysfunction [tw] OR renal dysfunction [tw] OR kidney impairment [tw] OR renal impairment [tw] OR kidney insufficiency [tw] OR renal insufficiency [tw] OR ESRD [tw] OR CKD [tw] OR kidney replacement therap* [tw] OR renal replacement therap* [tw] OR dialy* [tw] OR hemodialysis [tw] OR haemodialysis [tw] OR hematodialysis [tw] OR kidney transplantation [tw] OR renal transplantation [tw] OR kidney grafting [tw]

#2 atrial fibrillation [mh]

OR atrial fibrillation [tw] OR auricular fibrillation [tw] OR atrium fibrillation [tw]

#3 anticoagulants [mh] OR warfarin [tw]

OR anticoag* [tw] OR anti-coag* [tw] OR vitamin K antagonist [tw] OR VKA [tw] OR antithrombo* [tw] OR anti-thrombo* [tw] OR warfarin [mh] OR indirect thrombin inhibitor [tw] OR Coumadin* [tw] OR coumarin* [tw] OR dabigatran [tw] OR factor Xa inhibitor [tw] OR rivaroxaban [tw] OR apixaban [tw]

#1 AND #2 AND #3

### Embase

#1 ‘end stage renal disease’/exp OR ‘chronic kidney failure’/exp OR ‘renal replacement therapy’/exp OR ‘kidney transplantation’/exp

OR ‘kidney disease’:ab,ti OR ‘renal disease’:ab,ti OR ‘kidney failure’:ab,ti OR ‘renal failure’:ab,ti OR ‘kidney dysfunction’:ab,ti OR ‘renal dysfunction’:ab,ti OR ‘kidney impairment’:ab,ti OR ‘renal impairment’:ab,ti OR ‘kidney insufficiency’:ab,ti OR ‘renal insufficiency’:ab,ti OR esrd:ab,ti OR ckd:ab,ti OR (‘kidney replacement’ NEXT/1 therap*):ab,ti OR (‘renal replacement’ NEXT/1 therap*):ab,ti OR dialy*:ab,ti OR hemodialysis:ab,ti OR haemodialysis:ab,ti OR hematodialysis:ab,ti OR ‘kidney transplantation’:ab,ti OR ‘renal transplantation’:ab,ti OR ‘kidney grafting’:ab,ti

#2 ‘atrial fibrillation’/exp

OR ‘atrial fibrillation’:ab,ti OR ‘auricular fibrillation’:ab,ti OR ‘atrium fibrillation’:ab,ti

#3 ‘anticoagulant agent’/exp OR ‘warfarin’/exp OR anticoag*:ab,ti OR (anti NEXT/1 coag*):ab,ti OR ‘vitamin k antagonist’:ab,ti OR vka:ab,ti OR antithrombo*:ab,ti OR (anti NEXT/1 thrombo*):ab,ti OR warfarin:ab,ti OR ‘indirect thrombin inhibitor’:ab,ti OR coumadine:ab,ti OR dabigatran:ab,ti OR ‘factor xa inhibitor’:ab,ti OR rivaroxaban:ab,ti OR apixaban:ab,ti

#1 AND #2 AND #3

### Cochrane library

ID Search

#1 MeSH descriptor: [Kidney Failure, Chronic] explode all trees

#2 MeSH descriptor: [Renal Insufficiency, Chronic] explode all trees

#3 “kidney disease” or “renal disease” or “kidney failure” or “renal failure” or “kidney dysfunction” or “renal dysfunction” or “kidney impairment” or “renal impairment” or “kidney insufficiency” or “renal insufficiency” or ESRD or CKD

#4 MeSH descriptor: [Renal Replacement Therapy] explode all trees

#5 MeSH descriptor: [Renal Dialysis] explode all trees

#6 MeSH descriptor: [Kidney Transplantation] explode all trees

#7 kidney replacement therap* or renal replacement therap* or dialy* or hemodialysis or haemodialysis or hematodialysis or “kidney transplantation” or “renal transplantation” or “kidney grafting”

#8 #1 or #2 or #3 or #4 or #5 or #6 or #7

#9 MeSH descriptor: [Atrial Fibrillation] explode all trees

#10 “atrial fibrillation” or “auricular fibrillation” or “atrium fibrillation”

#11 #9 or #10

#12 MeSH descriptor: [Anticoagulants] explode all trees

#13 MeSH descriptor: [Warfarin] explode all trees

#14 anticoag* or anti-coag* or “vitamin K antagonist” or VKA or antithrombo* or anti-thrombo* or warfarin or “indirect thrombin inhibitor” or coumadine or dabigatran or “factor Xa inhibitor” or rivaroxaban or apixaban

#15 #12 or #13 or #14

#16 #8 and #11 and #15

**Table 3:** Stroke, bleeding and mortality outcomes reported in included studies

Author Year	Adjustment for confounding	Outcome	Outcome definition	Study groups	Number of patients with ESRD and AF	Number of outcome events	Rates per 100 PY (95 % CI)	Adjusted HR (95 % CI)
Chan 2009^e^ [30]	Adjusted for and PS matched on: CHADS_2_, gender, race, Charlson comorbidity index, entry date, access, body mass index, facility standardized mortality ratio, cardiovascular drugs, dialysis adequacy, baseline lab values, heparin dosage and heparin regimen	Any stroke	Hospitalization and death for stroke and TIAs identified from diagnoses obtained from hospital discharge summaries and cause of death from medical records	W (warfarin) C (no warfarin)	746 925	NR NR	7.1 (5.7, 8.7) 2.9 (2.0, 4.4)	1.74 (1.11, 2.72)^c^
		Ischemic stroke		W (warfarin) C (no warfarin)	746 925	NR NR	NR NR	1.81 (1.12, 2.92)
		Hemorrhagic stroke		W (warfarin) C (no warfarin)	746 925	NR NR	NR NR	2.22 (1.01, 4.91)
		Major bleeding	Hospitalization for bleeding	W (warfarin) C (no warfarin)	746 925	97 107	0.09 (NR) 0.07 (NR)	1.04 (0.73, 1.46)
		All-cause mortality	-	W (warfarin) C (no warfarin)	746 925	333 425	27.4 (NR) 25.7 (NR)	1.10 (0.93, 1.30)
Lai 2009^e^ [32]	Adjusted for: gender, age, GFR, hemodialysis/transplant, aspirin, stroke history, heart and artery disease, smoking, hypertension, diabetes, dyslipidemia	Ischemic stroke	Thromboembolic stroke	W (warfarin)	HD: 51; GFR: 78	HD: 5; GFR: 8	HD: 10 %^b^ GFR: 10 %	NR
				C (no warfarin)	HD: 42; GFR: 54	HD: 16 GFR: 20	HD: 38 % GFR: 37 %	
Wizemann 2010 [33]	Adjusted for: age category, sex, race, years with ESRD, study phase, history of stroke, comorbid conditions, permanent pacemaker implanted, history of cardiac arrest, left ventricular hypertrophy, valvular heart disease	Any stroke	Hospitalized for stroke or if they died with cause of death listed as ‘cerebrovascular accident (including intracranial hemorrhage)	W (warfarin) C (no warfarin)	509 2736	NR NR	NR NR	age ≤ 65: 1.29 (0.45, 3.68) age 66–75: 1.35 (0.69, 2.63) age > 75 years: 2.17 (1.04, 4.53)
Winkelmayer 2011 [22]	PS matched on: age, gender, race, state, dialysis vintage, dialysis type, comorbidity, vascular access surgery, length of stay, number of hospital days in prior year, number of medications used in prior year, H_2_ blocker or proton pump inhibitor use, prior nursing home stay, hematocrit, erythropoietin	Any stroke	Ischemic and hemorrhagic stroke identified by ICD-9	W (warfarin) C (no warfarin)	237 948	38 150	9.7 8.7	1.08^c^ (0.76, 1.55)
		Ischemic stroke	ICD-9: 433.x1, 434.x1, 436	W (warfarin) C (no warfarin)	237 948	29 135	7.4 7.8	0.92^c^ (0.61, 1.37)
		Hemorrhagic stroke	ICD-9: 430–432	W (warfarin) C (no warfarin)	237 948	11 121	2.6 7.5	2.38 (1.15, 4.96)
		Gastrointestinal bleed	Previously validated claims-based algorithm	W (warfarin) C (no warfarin)	237 948	48 216	13.4 13.6	0.96^c^ (0.70, 1.31)
		All-cause mortality	-	W (warfarin) C (no warfarin)	237 948	181 750	42.9 40.2	1.06^c^ (0.90, 1.24)
Olesen 2012 [23]	Adjusted for: changes in renal status or antithrombotic treatment during follow up, risk factors in the CHA_2_DS_2_-VASc score, antithrombotic treatment, year of inclusion	Any stroke	Hospitalization or death from stroke or systemic thromboembolism (peripheral-artery embolism, ischemic stroke and transient ischemic attack)	W (warfarin) C (no warfarin)	NR NR	NR NR	NR NR	0.44 (0.26, 0.74)
		Major bleeding	Bleeding (gastrointestinal, intracranial, urinary tract and airway bleeding)	W (warfarin) C (no warfarin)	NR NR	NR NR	NR NR	1.27 (0.91, 1.77)
Khalid 2013 [34]	PS adjusted for: age, gender, race, fresh frozen plasma, vitamin K, Charlson comorbidity index, cancer, INR, TTR, heart failure, aspirin, NSAIDs and clopidogrel use, ICU stay, blood transfusions	Ischemic stroke	thromboembolism	W (restarted warfarin) C (did not restart warfarin)	34 62	NR NR	NR NR	0.44 (0.27, 0.73)
		Gastrointestinal bleed	NR	W (restarted warfarin) C (did not restart warfarin)	34 62	NR NR	NR NR	1.72 (1.29, 2.30)
		All-cause mortality	-	W (restarted warfarin) C (did not restart warfarin)	34 62	NR NR	NR NR	0.22 (0.13, 0.40)
Wakasugi 2014 [29]	Adjusted for: CHADS_2_ score; PS matched on: age, gender, BMI, duration of dialysis, cause of ESRD, vascular access, medical history, medication, comorbidity, blood pressure, lab data, mobility	Ischemic stroke	New ischemic stroke (fatal or nonfatal) not including TIA	W (warfarin) C (no warfarin)	28 32	8 5	14.8 (6.4, 29.2) 8.9 (2.9, 20.8)	3.36^c^ (0.67, 16.66)
		Major bleeding	fatal bleeding or bleeding that required hospitalization	W (warfarin) C (no warfarin)	28 32	3 4	5.3 (1.1, 15.5) 6.6 (1.8, 17.0)	0.85^d^ (0.19, 3.64)
		All-cause mortality	-	W (warfarin) C (no warfarin)	28 32	9 9	14.2 (6.5, 26.9) 14.2 (6.5, 26.9)	1.00^d^ (0.40, 2.52)
Bonde 2014 [24]	Adjusted for: aspirin treatment, stroke/TE comorbidity, concomitant medication, CHA_2_DS_2_-VASc score, bleeding comorbidity, HAS-BLED score	Any stroke	Hospitalization/death from stroke/TE (i.e., peripheral arterial embolism, ischemic stroke and transient ischemic attack)	W (warfarin) C (no warfarin)	260 882	41^a,f^ 186^a,f^	NR NR	NR
		All-cause mortality	-	W (warfarin) C (no warfarin)	NR NR	NR NR	NR NR	Low/intermediate risk: 1.36 (0.96, 1.94)
								High risk: 0.85 (0.72, 0.99)
Carrero 2014 [25]	Adjusted for and PS matched on: age, sex, eGFR, comorbidities, patient presentation characteristics at admission, hospital course, discharge medication, center effect, left ventricular ejection fraction	Ischemic stroke	Hospitalizations due to ischemic stroke identified from claims	W (warfarin) C (no warfarin)	66 412	0 16	NR NR	MF
		Major bleeding	Readmission due to hemorrhagic stroke, gastrointestinal bleeding, bleeding causing anemia identified from claims	W (warfarin) C (no warfarin)	66 412	4 34	9.1 13.5	0.46^c^ (0.11, 1.89)
		All-cause mortality	-	W (warfarin) C (no warfarin)	66 412	32 222	NR NR	0.72 (0.46, 1.14)
Chen 2014 [26]	Adjusted for and PS matched on: age, gender, dialysis method, diabetes, risk factors, comorbidities, concomitant medication	Any stroke	Ischemic stroke, transient ischemic accident or hemorrhagic stroke identified by ICD9 codes	W (warfarin) C (no warfarin)	294 (250 analyzed) 2983 (250 analyzed)	NR NR	5.1%^b^ 6.6 %	1.017^c^ (0.673, 1.537)
Friberg 2014^e^ [13]	Adjusted for: renal failure, age, sex, year of inclusion, duration since first AF diagnosis, previous thrombo-embolism, venous thrombo-embolism, intracranial bleeding, anaemia, coagulopathy or platelet defect, vascular disease, heart failure, pericarditis, other valvular disease, pacemaker, comorbidity, baseline use of medication	Ischemic stroke	Principal or first secondary diagnosis code for ischemic stroke	W (warfarin) C (no warfarin)	3766 9669	NR NR	2.7 (2.3, 3.1) 4.6 (4.2, 4.9)	0.687 (0.584, 0.807)^h^
		Any stroke	Codes related to arterial or venous thromboembolism regardless of coding position	W (warfarin) C (no warfarin)	3766 9669	NR NR	6.2 (5.6, 6.8) 9.2 (8.7, 9.8)	0.779 (0.697, 0.870)^h^
		Intracranial bleeding	Principal or first secondary diagnosis codes for haemorrhage	W (warfarin) C (no warfarin)	3766 9669	NR NR	1.0 (0.8, 1.2) 0.7 (0.6, 0.9)	1.557 (1.149, 2.110)^h^
		Any bleeding	Codes related to intracranial, gastrointestinal and other bleeding	W (warfarin) C (no warfarin)	3766 9669	NR NR	9.6 (8.9, 10.4) 9.8 (9.3, 10.4)	1.096 (0.995, 1.206)^h^
		All-cause mortality	-	W (warfarin) C (no warfarin)	3766 9669	NR NR	24.7 (23.5, 25.8) 41.7 (40.7, 42.8)	0.747 (0.708, 0.788)^h^
Shah 2014 [27]	PS adjusted for: age, sex, specific components of CHADS_2_ or HAS-BLED score	Ischemic stroke	ischemic cerebrovascular disease including transient ischemic attack (TIA) and retinal infarct	W (warfarin) C (no warfarin)	756 870	52 55	3.37 (NR) 2.91 (NR)	1.17^c^ (0.79, 1.75)
		Any bleeding	intracerebral bleeding, gastrointestinal bleeding, intraocular bleeding, hematuria and unspecified location of bleeding	W (warfarin) C (no warfarin)	756 870	149 126	10.88 (NR) 7.31 (NR)	1.41^c^ (1.09, 1.81)
Genovesi 2015 [31]	Adjusted for: age and dialytic age, gender, antiplatelet therapy and hypertension status at recruitment, permanent AF and bleedings/haemorrhagic strokes, diabetes, ischaemic stroke, ischaemic heart disease and heart failure as time-dependent covariates	Any stroke	cerebrovascular event defined as ischemic or hemorrhagic by computed tomographic scan or nuclear magnetic resonance	W (warfarin) C (no warfarin)	134 156	8 9	3.7 (NR) 3.7 (NR)	0.12 (0.00, 3.59)
		Major bleeding	hemorrhagic episode requiring hospitalization or blood transfusion, or causing a hemoglobin plasma level reduction >2 g/dl	W (warfarin) C (no warfarin)	134 156	38 29	17.6 (NR) 11.8 (NR)	3.96 (1.15, 13.68)
		All-cause mortality	-	W (warfarin) C (no warfarin)	134 156	51 64	NR NR	0.96 (0.59, 1.56)
Chan KE 2015^e^ [20]	Adjusted for: age, sex, race, diabetes, vintage, catheter vascular access, blood pressure, albumin, hemoglobin, thrombocytopenia, erythropoietin dose, heparin dose, antiplatelet use, Charlson comorbidity score, bleeding index score, recent minor or major bleeding event	Ischemic stroke	embolic stroke or arterial embolism, within 2 years of medication initiation	W (warfarin) A (aspirin) D (dabigatran) R (rivaroxaban)	8064 6018 281 244	244 168 13 8	6.2 (NR) 5.0 (NR) 10.6 (NR) 11.2 (NR)	W vs. A: 1.23^g^ (1.01, 1.52) W vs. D: 1.71^g^ (0.97, 2.99) W vs R: 0.55^g^ (0.27, 1.12)
		Major bleeding	a hemorrhagic event resulting in hospitalization or death	W (warfarin) A (aspirin) D (dabigatran) R (rivaroxaban)	8064 6018 281 244	NR NR NR NR	47.1 (NR) 35.9 (NR) 83.1 (NR) 68.4 (NR)	W vs. A: 1.28^g^ (1.19, 1.39) W vs. D: 0.68^g^ (0.55, 0.83) W vs. R: 0.72^g^ (0.55, 0.97)
Chan PH 2015 [21]	Adjusted for: age, gender, hypertension, diabetes, smoker, heart failure, coronary artery disease, prior stroke/TIA, prior ICH, CHA_2_DS_2_-VASc, HAS-BLED	Ischemic stroke	a neurological deficit of sudden onset that persisted for more than 24 h in the absence of primary hemorrhage or other cause and confirmed by CT or MRI	W (warfarin) A (aspirin) C (no antithrombotic therapy)	67 86 118	0 9 11	NR NR NR	T vs. A: 0.16 (0.04, 0.66) T vs. C: 0.19 (0.06, 0.65)
Shen 2015 [28]	Included in inverse-probability treatment weighting analysis: age, sex, race, ethnicity, dialysis vintage, geographic location, comorbid conditions, indicators of health services use, baseline medication use, AF diagnosis characteristics	Any stroke	Any stroke or stroke death identified from claims-based algorithms	W (warfarin) C (no warfarin)	1838 10446	116 765	4.4 (NR) 5.3 (NR)	0.83^c^ (0.61, 1.12)
		Ischemic stroke	Identified from claims-based algorithms	W (warfarin) C (no warfarin)	1838 10446	63 503	2.3 (NR) 3.4 (NR)	0.68^c^ (0.47, 0.99)
		Gastrointestinal bleeding	events requiring hospitalization or with gastrointestinal bleeding as reported cause of death	W (warfarin) C (no warfarin)	1838 10446	153 833	5.9 (NR) 5.9 (NR)	1.00^c^ (0.69, 1.44)
		All-cause mortality	-	W (warfarin) C (no warfarin)	1838 10446	832 4595	33.0 (NR) 32.5 (NR)	1.01^c^ (0.92, 1.11)
Wang 2015 [14]	Adjusted for: cerebrovascular disease, congestive heart failure, body mass index, age, warfarin and aspirin, beta-blocker, ischaemic heart disease, peripheral vascular disease	Any stroke	Ischemic stroke and other arterial embolism	W (warfarin) C (no warfarin)	59 82	8 11	13.6%^b^ 13.4 %	1.01 (0.380, 2.70)^h^
		Ischemic stroke	focal neurological deficit lasting >24 h with radiological evidence on computed tomography or MRI	W (warfarin) C (no warfarin)	59 82	5 10	8.5%^b^ 12.2 %	0.667 (0.215, 2.06)^h^
		Any bleed	Intracranial bleed required radiological confirmation, while gastrointestinal, dialysis site and other bleeds required having a blood transfusion to be counted	W (warfarin) C (no warfarin)	59 82	22 24	37.3 %^b^ 29.3 %	1.44 (0.706, 2.92)^h^
		All-cause mortality	-	W (warfarin) C (no warfarin)	59 82	44 64	74.6%^b^ 78.0 %	0.825 (0.376, 1.81)^h^
Yodogawa 2015 [35]	Adjusted for: CHADS_2_ score	Any stroke	First hospital admission for stroke	W (warfarin) C (no warfarin)	30 54	2 5	NR NR	1.07 (0.20, 5.74)
Findlay 2016 [15]	No adjustment	Any stroke	Clinical diagnosis of stroke, presence of ischemic or hemorrhagic stroke on brain imaging, any stroke-related death	W (warfarin) C (no warfarin)	118 175	17 20	14.4 %^b^ 11.4 %	1.024^h^ (0.536, 1.959)^h^
		All-cause mortality	-	W (warfarin) C (no warfarin)	118 175	NR NR	NR NR	0.671^h^ (0.505, 0.891)^h^
Tanaka 2016 [16]	Adjusted for: age, sex, ACE/ARB, diabetes, history of CAD, heart failure, AD, eGFR, β-blocker, hemoglobin, calcium levels, phosphate levels and history of cerebral infarction and ICH	All-cause mortality	-	W (warfarin) C (no warfarin)	46 47	11 10	23.9%^b^ 21.3 %	0.7117^h^ (0.2475, 2.0463)^h^

*AF* atrial fibrillation, *HD* hemodialysis, *PD* peritoneal dialysis, *CKD* chronic kidney disease, *ESRD* end stage renal disease, *RRT* renal replacement therapy, *CI* confidence interval, *NR* not reported, *MF* model failed to converge, *CAD* coronary artery disease, *ICH* intracranial hemorrhage, *GFR* glomerular filtration rate, *AD* aortic disease

^a^Data were abstracted from online supplement

^b^Reference paper reported cumulative proportion instead of rates

^c^Effect measures listed in the table were from the ITT analysis or propensity score adjusted/matched analysis

^d^Reference table reported unadjusted HR

^e^Quality of warfarin treatment (i.e. INR or TTR) information provided

^f^Numbers were combined from CHA_2_DS_2_-VASc score = 0, = 1, and > = 2 subgroups

^g^ Reference paper reported RR instead of HR. Effect measure and 95 % CI was calculated by taking the reciprocal of the reported RR

^h^ Effect measure and/or 95 % CIs were obtained from personal communication with the study author

**Table 4:** Risk of bias assessment of included studies

Included Study	Judgment	Description
Chan 2009 [30]		
Bias due to *confounding*	Moderate	Analyses were adjusted for risk factors for stroke. These critically important domains were adjusted for using Cox regression analysis, and confirmed by propensity score adjusted analysis.
Bias in *selection of participants* into the study	Serious	Study “excluded patients with <90 d of study enrollment” so there may be some selection bias. “Patient outcomes were followed from the date of analysis initiation”, and drug exposure status was determined in the first 90 days of dialysis. Start of follow up and start of intervention do not coincide for all participants.
Bias in *measurement of interventions*	Low	Intervention status well defined and based on information collected at the time of intervention.
Bias due to *departures from intended interventions*	Low	The primary analysis was intention-to-treat whereby patients were not re-classified, two validation analyses with censoring and time-varying Cox model were used to account for departures from intended interventions. “Similar results were noted when patients were censored when they changed their warfarin, clopidogrel or aspirin prescription after study enrollment.”
Bias due to *missing data*	Low	Variables were identified from computerized medical results, so data were reasonably complete.
Bias in *measurement of outcomes*	Low	Outcomes were identified from the diagnoses obtained from hospital discharge summaries or medical records. The methods of outcome assessment were comparable across intervention groups.
Bias in *selection of the reported results*	Moderate	The outcome measurements and analyses were clearly defined. There is no clear indication of selection of the reported analysis from among multiple analyses or multiple subgroups.
Lai 2009 [32]		
Bias due to *confounding*	Critical	For the stroke outcome, study did not adjust for congestive heart failure. For the bleeding outcome, study did not adjust for liver disease, alcohol use and bleeding history. Adjusted analysis was not applied in comparing incidence of stroke and major bleeding episodes. Confounding inherently not controllable.
Bias in *selection of participants* into the study	Serious	All participants who would have been eligible for the target trial were included (“no patients were excluded from the analysis”). Study included prevalent users, so a potential important amount of follow-up time is missing for prevalent users from analyses.
Bias in *measurement of interventions*	Serious	Intervention status well defined but the intervention status was based on current use vs no use determined retrospectively. It may be determined in a way that could have been affected by knowledge of the outcome.
Bias due to *departures from intended interventions*	Serious	Bias due to departure from the intended intervention is expected, and is not adjusted for in the analyses.
Bias due to *missing data*	No information	Medical charts of eligible patients treated in a medical center were reviewed. No information about loss to follow up or missing data.
Bias in *measurement of outcomes*	Low	Outcomes were identified from the medical charts. The methods of outcome assessment were comparable across intervention groups.
Bias in *selection of the reported results*	Moderate	The outcome measurements and analyses were clearly defined. There is no clear indication of selection of the reported analysis from among multiple analyses or multiple subgroups.
Wizemann 2010 [33]		
Bias due to *confounding*	Serious	Study adjusted for risk factors in Cox regression analysis, but not confirmed by additional analyses.
Bias in *selection of participants* into the study	Critical	Study included patients who had pre-existing (i.e. a history of) AF at enrollment and patients who subsequently hospitalized with the diagnosis of AF. Study included prevalent users, so a substantial amount of follow-up time is missing for prevalent users from analyses.
Bias in *measurement of interventions*	Moderate	Intervention is well defined, but some aspects of the assignments of intervention status were determined retrospectively since self-reported medication use status may be subject to recall bias.
Bias due to *departures from intended interventions*	No information	Study did not describe the analytical method in detail.
Bias due to *missing data*	No information	Study was based on an international, observational study of HD facilities. No information about loss to follow up or missing data.
Bias in *measurement of outcomes*	Moderate	Outcomes were identified from hospitalization or death records. The outcome measure may be minimally influenced by knowledge of the intervention received by study participants, since study was retrospective in nature.
Bias in *selection of the reported results*	Serious	The outcome measurements and analyses were not clearly described. Study reported outcomes by age subgroups rather than the entire cohort. Study highlighted significant result in the highest risk age subgroup in the abstract.
Winkelmayer 2011 [22]		
Bias due to *confounding*	Moderate	Study adjusted for risk factors included in the CHADS_2_ and HAS-BLED score in the time-fixed Cox regression analysis, and confirmed using propensity score-adjusted analyses which yielded similar results.
Bias in *selection of participants* into the study	Low	All participants who would have been eligible for the target trial were included. Start of intervention and start of intervention coincide for all subjects (30 days after AF hospital discharge).
Bias in *measurement of interventions*	Low	Intervention is well defined, and based solely on information collected at the time of intervention in the regional hospital discharge abstract and drug claims databases.
Bias due to *departures from intended interventions*	Low	Study used intention-to-treat in which patients were “only censored for end of database” in the primary analysis. Study also used as-treated analysis which “censored patients at treatment cross-over”.
Bias due to *missing data*	Low	Study obtained data from the national patient registry and regional healthcare claims database which contains information on relevant ESRD patients. Data were reasonably complete.
Bias in *measurement of outcomes*	Low	Outcomes were identified using previously validated claims-based algorithm. The methods of outcome assessment were comparable across intervention groups.
Bias in *selection of the reported results*	Moderate	The outcome measurements and analyses were clearly defined. There is no clear indication of selection of the reported analysis from among multiple analyses or multiple subgroups.
Olesen 2012 [23]		
Bias due to *confounding*	Serious	Study adjusted for risk factors included in the CHA_2_DS_2_-VASc and HAS-BLED score in the time-dependent Cox regression analysis, but not confirmed by additional analyses.
Bias in *selection of participants* into the study	Low	All participants who would have been eligible for the target trial were included. Start of intervention and start of intervention coincide for all subjects. “The baseline assessment and follow-up period began 7 days after discharge.”
Bias in *measurement of interventions*	Low	Intervention is well defined, and based solely on information collected at the time of intervention in the national patient registry.
Bias due to *departures from intended interventions*	Serious	Study uses time-dependent analysis, so bias due to departures from intended interventions is expected. Study did not adjust for switches, co-intervention or problems with implementation fidelity in the analyses.
Bias due to *missing data*	Low	Study obtained data from the national patient registry which contains information on all residents. Data were reasonably complete.
Bias in *measurement of outcomes*	Low	Outcomes were identified using claims-based algorithm. The methods of outcome assessment were comparable across intervention groups.
Bias in *selection of the reported results*	Moderate	The outcome measurements and analyses were clearly defined. There is no clear indication of selection of the reported analysis from among multiple analyses or multiple subgroups.
Khalid 2013 [34]		
Bias due to *confounding*	Critical	Analyses did not adjust for important confounders such as hypertension, cerebrovascular disease, diabetes.
Bias in *selection of participants* into the study	Critical	Participants re-started warfarin after experiencing bleeding outcomes, so their risk profile may be different. Study included prevalent users, so a potential important amount of follow-up time is missing for prevalent users from analyses.
Bias in *measurement of interventions*	Moderate	Intervention was not well defined. Not clear when the start of intervention was.
Bias due to *departures from intended interventions*	Serious	Treatment could be discontinued due to experiencing bleeding outcomes. Study did not address bias due to departures from intended interventions.
Bias due to *missing data*	No information	
Bias in *measurement of outcomes*	Low	Intervention not well defined, but were “adjudicated by two blind reviewers”.
Bias in *selection of the reported results*	Moderate	The outcome measurements and analyses were clearly defined. There is no clear indication of selection of the reported analysis from among multiple analyses or multiple subgroups.
Wakasugi 2014 [29]		
Bias due to *confounding*	Critical	For the stroke outcome, analysis was adjusted for CHADS_2_ score and matched by propensity score. For the major bleeding and all-cause mortality outcomes, analyses were not adjusted.
Bias in *selection of participants* into the study	Serious	All participants who would have been eligible for the target trial were included. Study included prevalent users, so a potential important amount of follow-up time is missing for prevalent users from analyses.
Bias in *measurement of interventions*	Low	Intervention is well defined, and based solely on information collected at the time of intervention since study was conducted prospectively.
Bias due to *departures from intended interventions*	Low	“The primary analysis was intention-to-treat in which patients who started using warfarin after study enrollment were not reclassified”, so the specified comparison relates to initiation of intervention regardless of whether it is continued. To account for possible longitudinal changes in drug prescription over time, study also repeated the analysis in which patients were censored when warfarin use changed.
Bias due to *missing data*	No information	Medical charts of eligible patients treated in a medical center were reviewed. No information about loss to follow up or missing data.
Bias in *measurement of outcomes*	Low	Outcomes were identified from the medical charts. The methods of outcome assessment were comparable across intervention groups.
Bias in *selection of the reported results*	Moderate	The outcome measurements and analyses were clearly defined. There is no clear indication of selection of the reported analysis from among multiple analyses or multiple subgroups.
Bonde 2014 [24]		
Bias due to *confounding*	Serious	Analyses were adjusted for risk factors included in the CHA_2_DS_2_-VASc and HAS-BLED score with age as a continuous covariate. These critically important domains were adjusted for using Cox regression analysis, but not confirmed by additional analyses.
Bias in *selection of participants* into the study	Low	All participants who would have been eligible for the target trial were included. Start of follow up and start of intervention coincide for all subjects (follow-up began 7 days after discharge).
Bias in *measurement of interventions*	Moderate	Intervention is well defined, but assignment of intervention status by “dividing the number of tablets dispensed with the estimated daily dosage” was determined retrospectively.
Bias due to *departures from intended interventions*	Serious	“Treatment is often discontinued in terminal patients” so switches and discontinuation of treatment were apparent. Such departures from intended intervention may be a result of outcomes of interest. Study used time-dependent, as-treated Cox regression analysis, but did not adjust for such departures appropriately.
Bias due to *missing data*	Low	Accurate data on all patients actively treated for end-stage CKD with RRT recorded in national registry, so data were reasonably complete.
Bias in *measurement of outcomes*	Low	Outcomes were identified from ICD-8 or ICD-10 codes and the same method of outcome assessment was applied across intervention groups.
Bias in *selection of the reported results*	Serious	Study stratified and analyzed results based on CHA_2_DS_2_-VASc score subgroups. Main result reported the high risk subgroup, which appears to be reported on the basis of significant result.
Carrero 2014 [25]		
Bias due to *confounding*	Moderate	Study accounted for the most important confounders and confirmed results by propensity score-matched analysis. Residual confounding is inherent in observational studies.
Bias in *selection of participants* into the study	Low	All participants who would have been eligible for the target trial were included. Start of intervention and start of intervention coincide for all subjects (starts with warfarin prescription at discharge).
Bias in *measurement of interventions*	Low	Intervention is well defined, and based solely on information collected at the time of intervention.
Bias due to *departures from intended interventions*	Low	“Warfarin use vs no warfarin use was considered as a time-fixed binary variable throughout the follow-up period.” Specified comparison relates to initiation of intervention regardless of whether it is continued as in intention-to-treat (ITT) analysis in target trial.
Bias due to *missing data*	Low	“The use of unique personal identification number for all Swedish citizens and continuously updated national registries on death date, cause of death and emigration allow a virtually complete follow-up” with no loss to follow up.
Bias in *measurement of outcomes*	Low	Outcomes were identified from the National Inpatient Registry and the same method of outcome assessment was applied across intervention groups.
Bias in *selection of the reported results*	Moderate	The outcome measurements and analyses were clearly defined. There is no clear indication of selection of the reported analysis from among multiple analyses or multiple subgroups.
Chen 2014 [26]		
Bias due to *confounding*	Moderate	Analyses were adjusted for risk factors for stroke. These critically important domains were adjusted for using propensity score matched analysis. Ischemic stroke/TIA history was excluded from the study population. Residual confounding is inherent in observational studies.
Bias in *selection of participants* into the study	Low	All participants who would have been eligible for the target trial were included. Start of intervention and start of intervention coincide for all subjects.
Bias in *measurement of interventions*	Low	Intervention status well defined and based on information collected at the time of intervention.
Bias due to *departures from intended interventions*	Low	The primary analysis was intention-to-treat, thus the specified comparison relates to initiation of intervention regardless of whether it is continued.
Bias due to *missing data*	Low	Variables were identified from the universal national health insurance program, so data were reasonably complete.
Bias in *measurement of outcomes*	Low	Outcomes were identified from the ICD diagnosis codes. The methods of outcome assessment were comparable across intervention groups.
Bias in *selection of the reported results*	Moderate	The outcome measurements and analyses were clearly defined. There is no clear indication of selection of the reported analysis from among multiple analyses or multiple subgroups.
Friberg 2014 [13]		
Bias due to *confounding*	Moderate	Analyses were adjusted for risk factors for stroke and bleeding. These critically important domains were adjusted for using propensity score adjusted analysis. Residual confounding is inherent in observational studies.
Bias in *selection of participants* into the study	Serious	Study included prevalent users (taking warfarin at baseline i.e. 5 months before, and up to 1 month after the index AF diagnosis), so an important amount of follow-up time is missing for prevalent users from analyses. Start of follow up and start of intervention do not coincide for all subjects (time at risk for survival analyses started on Day +14 after index AF diagnosis).
Bias in *measurement of interventions*	Low	Intervention status well defined and based on information collected at the time of intervention.
Bias due to *departures from intended interventions*	Low	The primary analysis was intention-to-treat, thus the specified comparison relates to initiation of intervention regardless of whether it is continued.
Bias due to *missing data*	Low	Variables were identified from the national patient registry, so data were reasonably complete.
Bias in *measurement of outcomes*	Low	Outcomes were identified from the ICD diagnosis codes. The methods of outcome assessment were comparable across intervention groups.
Bias in *selection of the reported results*	Moderate	The outcome measurements and analyses were clearly defined. There is no clear indication of selection of the reported analysis from among multiple analyses or multiple subgroups.
Shah 2014 [27]		
Bias due to *confounding*	Moderate	Study adjusted for risk factors included in the CHADS_2_ and HAS-BLED score in the time-fixed Cox regression analysis, and confirmed using propensity score-adjusted analyses which yielded similar results.
Bias in *selection of participants* into the study	Low	All participants who would have been eligible for the target trial were included. Start of follow up and start of intervention coincide for all subjects (30 days after AF hospital discharge).
Bias in *measurement of interventions*	Low	Intervention is well defined, and based solely on information collected at the time of intervention in the regional hospital discharge abstract and drug claims databases.
Bias due to *departures from intended interventions*	Low	Study uses time-fixed, intention-to-treat analysis, so the specified comparison relates to initiation of intervention regardless of whether it is continued.
Bias due to *missing data*	Low	Study obtained data from the regional hospital and drug claims databases which contains information on all residents in the region. Data were reasonably complete.
Bias in *measurement of outcomes*	Low	Outcomes were identified using claims-based algorithm. The methods of outcome assessment were comparable across intervention groups.
Bias in *selection of the reported results*	Moderate	The outcome measurements and analyses were clearly defined. There is no clear indication of selection of the reported analysis from among multiple analyses or multiple subgroups.
Genovesi 2015 [31]		
Bias due to *confounding*	Moderate	Analyses were adjusted for risk factors for stroke and bleeding. These critically important domains were adjusted for using multivariate Cox regression analysis followed by propensity score matched analysis. Residual confounding is inherent in observational studies.
Bias in *selection of participants* into the study	Serious	Study included prevalent users (taking anticoagulant at recruitment) and incident users (starting anticoagulant within 2 weeks following recruitment), so a potential important amount of follow-up time is missing for prevalent users from analyses.
Bias in *measurement of interventions*	Low	Intervention status well defined and based on information collected at the time of intervention.
Bias due to *departures from intended interventions*	Low	The primary analysis was intention-to-treat, thus the specified comparison relates to initiation of intervention regardless of whether it is continued.
Bias due to *missing data*	No information	Patients from 10 hemodialysis center were prospectively followed-up for 2 years. No information about loss to follow up or missing data.
Bias in *measurement of outcomes*	Low	Outcomes were identified from medical charts using clinical diagnosis criteria. The methods of outcome assessment were comparable across intervention groups.
Bias in *selection of the reported results*	Moderate	The outcome measurements and analyses were clearly defined. There is no clear indication of selection of the reported analysis from among multiple analyses or multiple subgroups.
Chan KE 2015 [20]		
Bias due to *confounding*	Moderate	Study accounted for the most important confounders and “mitigated potential bias from unmeasured factors by performing a matched analysis which supported the main findings of the study”. Residual confounding is inherent in observational studies.
Bias in *selection of participants* into the study	Low	All participants who would have been eligible for the target trial were included. Start of follow up and start of intervention coincide for all subjects (followed from the time the initiated anticoagulant treatment de novo).
Bias in *measurement of interventions*	Low	Intervention status well defined and based on information collected at the time of intervention.
Bias due to *departures from intended interventions*	Serious	Patient was censored with discontinuation of treatment medications. Study used Poisson regression which does not take into account of the potentially varying rate (hazard) ratio for the effect of intervention.
Bias due to *missing data*	Low	“All subjects registered in the database are followed longitudinally, where data parameters are actively collected,” so data were reasonably complete.
Bias in *measurement of outcomes*	Low	The methods of outcome assessment were comparable across intervention groups. Bleeding outcomes were adjudicated retrospectively, but “the adjudicator was blinded to patient, treatment group and outcome”.
Bias in *selection of the reported results*	Moderate	The outcome measurements and analyses were clearly defined. There is no clear indication of selection of the reported analysis from among multiple analyses or multiple subgroups.
Chan PH 2015 [21]		
Bias due to *confounding*	Serious	Study did not list variables adjusted in the multivariate Cox regression analysis. Results were not confirmed by additional analyses.
Bias in *selection of participants* into the study	Critical	Study included patients who had pre-existing AF at enrollment. Study included prevalent users, so a substantial amount of follow-up time is missing for prevalent users from analyses. Study excluded patients with incomplete follow-up data, so there may be some selection bias.
Bias in *measurement of interventions*	Low	Intervention is well defined, and based on medical records and discharge summaries at the time of medication prescription.
Bias due to *departures from intended interventions*	Serious	Study compared current users vs non-users. Bias due to departure from the intended intervention is expected, but not adjusted for in the analysis.
Bias due to *missing data*	Low	Study data were retrieved from the medical records and discharge summaries from the territory-wide information network of all public hospitals in the region. Study excluded patients with incomplete clinical and/or follow-up data, so data were reasonably complete.
Bias in *measurement of outcomes*	Moderate	Outcomes were identified from medical records. The outcome measure may be minimally influenced by knowledge of the intervention received by study participants, since study was retrospective in nature.
Bias in *selection of the reported results*	Moderate	The statistical analyses were not clearly described. There is no clear indication of selection of the reported analysis from among multiple analyses or multiple subgroups.
Shen 2015 [28]		
Bias due to *confounding*	Moderate	Study used Cox regression analysis in the propensity score matched analysis. History of stroke was not included in the analysis. Unmeasured confounding is inherent in observational studies.
Bias in *selection of participants* into the study	Low	All participants who would have been eligible for the target trial were included. Start of intervention and start of intervention coincide for all subjects (30 days after AF hospital discharge).
Bias in *measurement of interventions*	Low	Intervention is well defined, and based solely on information collected at the time of intervention in the national patient registry.
Bias due to *departures from intended interventions*	Low	Study used intention-to-treat and as-treated analyses, and adjusted for selection bias using inverse probability of treatment and censoring-weighted (IPTW) analysis.
Bias due to *missing data*	Low	Study obtained data from the national patient registry which contain information on all ESRD patients. Data were reasonably complete.
Bias in *measurement of outcomes*	Low	Outcomes were identified using previously validated claims-based algorithm. The methods of outcome assessment were comparable across intervention groups.
Bias in *selection of the reported results*	Moderate	The outcome measurements and analyses were clearly defined. There is no clear indication of selection of the reported analysis from among multiple analyses or multiple subgroups.
Wang 2015 [14]		
Bias due to *confounding*	Serious	Study reported adjusted multivariate analyses for outcomes (all predictors with P < 0.10 listed). Confounding inherently not controllable. Results not confirmed by additional analyses.
Bias in *selection of participants* into the study	Critical	Study included prevalent users, so a substantial amount of follow-up time is missing for prevalent users from analyses.
Bias in *measurement of interventions*	Low	Intervention is well defined, and based on medical records and discharge summaries at the time of medication prescription.
Bias due to *departures from intended interventions*	Serious	Study compared current users vs non-users. Bias due to departure from the intended intervention is expected, but not adjusted for in the analysis.
Bias due to *missing data*	No information	Study data were retrieved from the medical records in a hospital. No information about loss to follow up or missing data.
Bias in *measurement of outcomes*	Moderate	Outcomes were identified from medical records. The outcome measure may be minimally influenced by knowledge of the intervention received by study participants, since study was retrospective in nature.
Bias in *selection of the reported results*	Serious	Study analyzed multiple outcomes and only reported results for predictors with significant results (P < 0.10) rather than a priori model. There is a high risk of selective reporting from among multiple analyses.
Yodogawa 2015 [35]		
Bias due to *confounding*	Serious	Study adjusted for CHADS_2_ in Cox regression analysis, but not confirmed by additional analyses.
Bias in *selection of participants* into the study	Critical	Study included patients who had pre-existing AF at enrollment. Study included prevalent users, so a substantial amount of follow-up time is missing for prevalent users from analyses. Study excluded patients with <6 months life expectancy, so the association may be attenuated since the high risk patients were excluded from analysis.
Bias in *measurement of interventions*	Moderate	Intervention is well defined, but some aspects of the assignments of intervention status were determined retrospectively since self-reported medication use status may be subject to recall bias.
Bias due to *departures from intended interventions*	No information	Study did not describe the analytical method in detail.
Bias due to *missing data*	Low	Study was based on a retrospective, observational study on medical records of patients treated in a hospital. Study excluded patients without follow-up data, so data were reasonably complete.
Bias in *measurement of outcomes*	Moderate	Outcomes were identified from medical records. The outcome measure may be minimally influenced by knowledge of the intervention received by study participants, since study was retrospective in nature.
Bias in *selection of the reported results*	Moderate	The outcome measurements and analyses were not clearly described. There is no clear indication of selection of the reported analysis from among multiple analyses or multiple subgroups.
Findlay 2016 [15]		
Bias due to *confounding*	Critical	Study used Kaplan-Meier survival curve to analyze outcomes between warfarin and no warfarin group, and did not use adjusted analysis to control for confounding.
Bias in *selection of participants* into the study	Critical	Study included patients who had pre-existing AF at enrollment. Study included prevalent users, so a substantial amount of follow-up time is missing for prevalent users from analyses.
Bias in *measurement of interventions*	No information	Study did not provide information about measurement of interventions.
Bias due to *departures from intended interventions*	Critical	Study did not describe or adjust for any bias due to departures from intended interventions.
Bias due to *missing data*	No information	Study did not provide information about missing data.
Bias in *measurement of outcomes*	Moderate	Outcomes were identified from the electronic patient record, and was reviewed by 2 independent clinicians. The outcome measure may be minimally influenced by knowledge of the intervention received by study participants, since study was retrospective in nature.
Bias in *selection of the reported results*	Moderate	The outcome measurements and analyses were not clearly described. There is no clear indication of selection of the reported analysis from among multiple analyses or multiple subgroups.
Tanaka 2016 [16]		
Bias due to *confounding*	Critical	Study used Kaplan-Meier survival curve to analyze outcomes between warfarin and no warfarin group, and did not use adjusted analysis to control for confounding.
Bias in *selection of participants* into the study	Critical	Study included patients who had pre-existing AF at enrollment. Study included prevalent users, so a substantial amount of follow-up time is missing for prevalent users from analyses.
Bias in *measurement of interventions*	Low	Intervention is well defined, and based on records collected during the period of dialysis initiation. Since study was prospective, assume no recall bias.
Bias due to *departures from intended interventions*	Critical	Study did not describe or adjust for any bias due to departures from intended interventions.
Bias due to *missing data*	Low	Study excluded patients lost to follow-up, so data were reasonably complete.
Bias in *measurement of outcomes*	Moderate	Outcomes were identified from survey slips that were sent to the dialysis facilities. The outcome measure may be minimally influenced by knowledge of the intervention received by study participants.
Bias in *selection of the reported results*	Moderate	The outcome measurements and analyses were not clearly described.There is no clear indication of selection of the reported analysis from among multiple analyses or multiple subgroups.

## Abbreviations

AF: Atrial fibrillation
CI: Confidence interval
CKD: Chronic kidney disease
DOAC: Direct oral anticoagulants
ESRD: End stage renal disease
GFR: Glomerular filtration rate
HD: Hemodialysis
HR: Hazard ratio
INR: International normalized ratio
PD: Peritoneal dialysis
RR: Risk ratio
RRT: Renal replacement therapy
TTR: Time in therapeutic range
